# The Association of Gut Microbiota With Idiopathic Central Precocious Puberty in Girls

**DOI:** 10.3389/fendo.2019.00941

**Published:** 2020-01-22

**Authors:** Guoqing Dong, Jiyong Zhang, Zhenyu Yang, Xin Feng, Jianxu Li, Dongfang Li, Miao Huang, Yinhu Li, Minghui Qiu, Xiyan Lu, Peihui Liu, Yongmei Zeng, Ximing Xu, Xiaoping Luo, Wenkui Dai, Sitang Gong

**Affiliations:** ^1^Department of Pediatrics, Shenzhen Maternity and Child Healthcare Hospital, Shenzhen, China; ^2^School of Statistics and Data Science, NanKai University, Tianjin, China; ^3^Microbial Department, WeHealthGene Institute, Shenzhen, China; ^4^Department of Pediatrics, Tongji Hospital, Tongji Medical College, Huazhong University of Science and Technology, Wuhan, China; ^5^Joint Laboratory of Microecology and Children's Health, Shenzhen Children's Hospital and Shenzhen WeHealthGene Co. Ltd, Shenzhen, China; ^6^Department of Pediatrics, Guangzhou Women and Children's Medical Center, Guangzhou, China

**Keywords:** idiopathic central precocious puberty, gut microbota, short chain fatty acids, leptin, 16S rDNA sequencing

## Abstract

Idiopathic central precocious puberty (ICPP) is a relatively common condition in preadolescent girls, and its pathogenesis remains to be uncovered. A variety of studies have highlighted the association of gut microbiota (GM) with endocrine diseases, such as obesity, which is commonly associated with ICPP. However, the relationship between GM and ICPP remains unexplored. Feces samples were collected from 25 girls with ICPP (ICPP group) and 23 healthy girls (Control group). We applied 16S rDNA sequencing to compare the GM between two groups. The ICPP group had higher GM diversity and was enriched for several GM species, including *Ruminococcus gnavus, Ruminococcus callidus, Ruminococcus bromii, Roseburia inulinivorans, Coprococcus eutactus, Clostridium leptum*, and *Clostridium lactatifermentans*, which are known to be associated with obesity and are related to the production of short-chain fatty acids. Additionally, 36 candidate GM biomarkers for patients with ICPP screening were identified with high accuracy (AUC = 0.95, 95% CI 0.88 to 1). We observed that the GM of the ICPP group was enriched for the microbial functions of cell motility, signal transduction, and environmental adaptation. Positive correlations were also detected between *Fusobacterium* and follicle-stimulating hormone, and *Gemmiger* and luteinizing hormone. This study documents relationships between GM and ICPP, and the implication of these findings remains to be determined.

## Introduction

Central precocious puberty (CPP) results from earlier than expected maturation of the hypothalamic-pituitary-gonadal (HPG) axis during preadolescence (1). CPP is a relatively common pediatric endocrine condition, observed in 1 in 5,000 to 1 in 10,000 children (2). When CPP occurs in a patient without an identifiable predisposing condition, it is called idiopathic CPP (ICPP) (3). Girls are more likely to have ICPP than boys (5 to 10 times higher prevalence in girls than in boys) (4). ICPP triggers signs of sexual maturation earlier than typically expected, with the appearance of secondary sex characteristics before 8 and 9 years of age in girls and boys, respectively (5). The pathogenesis of ICPP remains to be explored.

Gut microbiota (GM), which is an aggregation of intestinal microorganisms, influences the human endocrine system through activation of the enteric nervous system via microbial metabolites (6, 7). Previous studies have found close associations between obesity and puberty (8). Body mass index (BMI) is positively related to the risk of precocious puberty (9), and a 1 kg gain in weight is believed to cause a 13 day advancement of menarchal age (10). Moreover, GM dysbiosis has been reported in patients with obesity, with an increase in Firmicutes and a reduction in Bacteroidetes, and an improved ability of GM to utilize energy in those with obesity (11). Given that girls with ICPP tend to be obese (12), we hypothesized that dysbiosis of GM in patients with ICPP might be similar to that in patients with obesity, and that GM might participate in the pathogenesis of ICPP.

In this study, we recruited 25 girls with ICPP (ICPP group) and 23 healthy girls (Control group) to investigate the GM characteristics in the patients with ICPP. Through the comparison of GM between these two groups, we aimed to: (1) find discrepancies of GM structure and function in patients with ICPP; (2) detect correlations between GM composition and clinical indicators; and (3) identify GM biomarkers for the early prediction of ICPP. This study will promote our understanding of the pathogenesis of ICPP from the perspective of GM.

## Methods

### Ethics Statement

The study was approved by the Shenzhen Maternity and Child Health Hospital Ethics under Committee number: (2018)216. All procedures were performed in accordance with relevant guidelines and regulations stipulated by the Ethics Committee of the Shenzhen Maternity and Child Healthcare Hospital. All of the children's parents provided written informed consent, and volunteered to allow their children to participate in this investigation.

### Sample Preparation

Patients with ICPP and healthy girls were recruited from the Shenzhen Maternity and Child Healthcare Hospital. Feces were collected in a pre-labeled sterile stool collection kit (ML-001A, Micosolution, Dayun Gene, China) by individual subjects at home. Feces collected in the kit were sent to the hospital within 12 h and then stored at −80°C. The girls who satisfied the following inclusion criteria for ICPP were enrolled: (1) younger than 8 years; (2) Tanner stage B2 or higher; (3) increased volume of the uterus and ovary; (4) peak luteinizing hormone (LH) >5.0 IU/L on the GnRH stimulation test (5, 13–15) AND one of following criteria: (i) the difference of bone age and chronological age above or close to 1 year; (ii) GnRH-stimulated peak LH-to-follicle-stimulating hormone (FSH) ratio above or close to 0.6; (iii) secondary sex characteristics obviously recognized. Patients were excluded from the study if they met the following criteria: (1) diagnosed with peripheral precocious puberty (PPP), or central precocious puberty caused by an organic lesion; (2) associated endocrine, gastrointestinal, metabolic disease (including obesity and diabetes, among others), mental disease, or hepatobiliary disease; or (3) exposed to antibiotics, or probiotics 2 months before feces collection. The enrolled healthy girls were selected according to the following criteria: (1) between 5 and 16 years of age; (2) girls younger than 8 years old did not have precocious puberty; and (3) there was no history of discomfort, disease, or medication history 1 month before feces collection.

### DNA Extraction, Library Construction, and Sequencing

Microbial genomic DNA was extracted from stool using the DNeasy Power Soil kit (Qiagen, Hilden, Germany) according to the manufacturer's protocols. A Qubit spectrophotometer was used to evaluate the quality of isolated DNA (Qubit, Thermo Fisher Scientific, Singapore). Universal primers (338F and 806R) were used to amplify the V3-V4 region of 16S rDNA from metagenomic DNA in feces (TransGenAP221-02, Peking). The PCR reaction conditions for amplification of DNA were as follows: initial denaturation at 95°C for 3 min, followed by 27 cycles of denaturation at 95°C for 30 s, annealing at 55°C for 30 s, and extension at 72°C for 30 s. Primer sets were modified with Illumina adapter regions for sequencing library construction (TruSeq DNA PCR-Free kit, Illumina, San Diego, CA, United States). The library was sequenced with 300 Paired-end sequencing strategy using the Miseq platform (Illumina, San Diego, CA, United States).

### Bioinformatics Analysis

To obtain high-quality reads, the raw reads were processed with the following steps: (1) filtering of primer sequences; (2) truncating of low quality reads (averaged base quality <20 within a 30 bp sliding window); (3) removing trimmed paired-reads if its length was <75% of the original length; and (4) discarding whole paired-reads if there were any ambiguous base calls. The high-quality reads were connected into tags, and the tags were clustered into Operational Taxonomic Units (OTUs) with 97% similarity using USEARCH (version 9.1.13) (16). Meanwhile, chimeric sequences were discarded by the same software (16). The OTUs were aligned to the Ribosomal Database Project (RDP) database (June 2016 version) using Naive Bayesian Classifier v2.2 for the annotation of genus and species under the same setting (default parameters, confidence level threshold = 0.8) (17). Non-metric multidimensional scaling (nMDS) analysis was applied to investigate the genus differences between the two groups using the “vegan” package in R (version 3.5.1) (18). The Shannon index was calculated also using the “vegan” package in R (version 3.5.1) (18).

For bacterial biomarker identification, a two-step method was adopted. First, the “rfcv” function of the random Forest R package (19) was applied to determine the optimal biomarker number and the “random forest” function to select these 36 bacterial biomarkers according to the Mean Decrease Gini value (Gini threshold = 0.187) based on all of the samples. Second, based on these 36 candidate biomarkers, a 5-fold cross-validation was employed for classification: All of the samples were randomly divided into five sets, four of which were employed for constructing a random forest model, and the remaining one was used to validate the model. The area under the operating characteristic curve (AUC) was calculated and visualized using the R package “pROC” (20). The previously described process was iterated five times with default parameters.

GM functions were predicted on the basis of 16S rDNA OTUs profiling using PICRUSt with default parameters (21). The relative abundance of KEGG orthology (KO) was calculated for each sample. KO enrichment on level II functional categories of the KEGG database was detected.

### Statistical Analysis

Power analysis was used to estimate the sample size (“pwr v1.2-2” package in R). Wilcoxon rank-sum test (“wilcox.test” package in R) was utilized to detect differentially enriched microbe (genus and species-level) and GM functional categories between ICPP and Control groups (FDR < 0.05). Non-parametric permutational multivariate analysis of variance (PERMANOVA) was conducted using the “vegan” package for assessing the driving factors of microbiota composition (FDR < 0.05) (18). A Spearman correlation approach was employed to investigate the relationships among GM structure (on genus-level), GM functional categories, and three clinical indicators. The method of Benjamini-Hochberg was adopted to adjust the results using “p.adjust” in R.

## Results

### Sample Characteristics and Data Output

**Figure 1:**
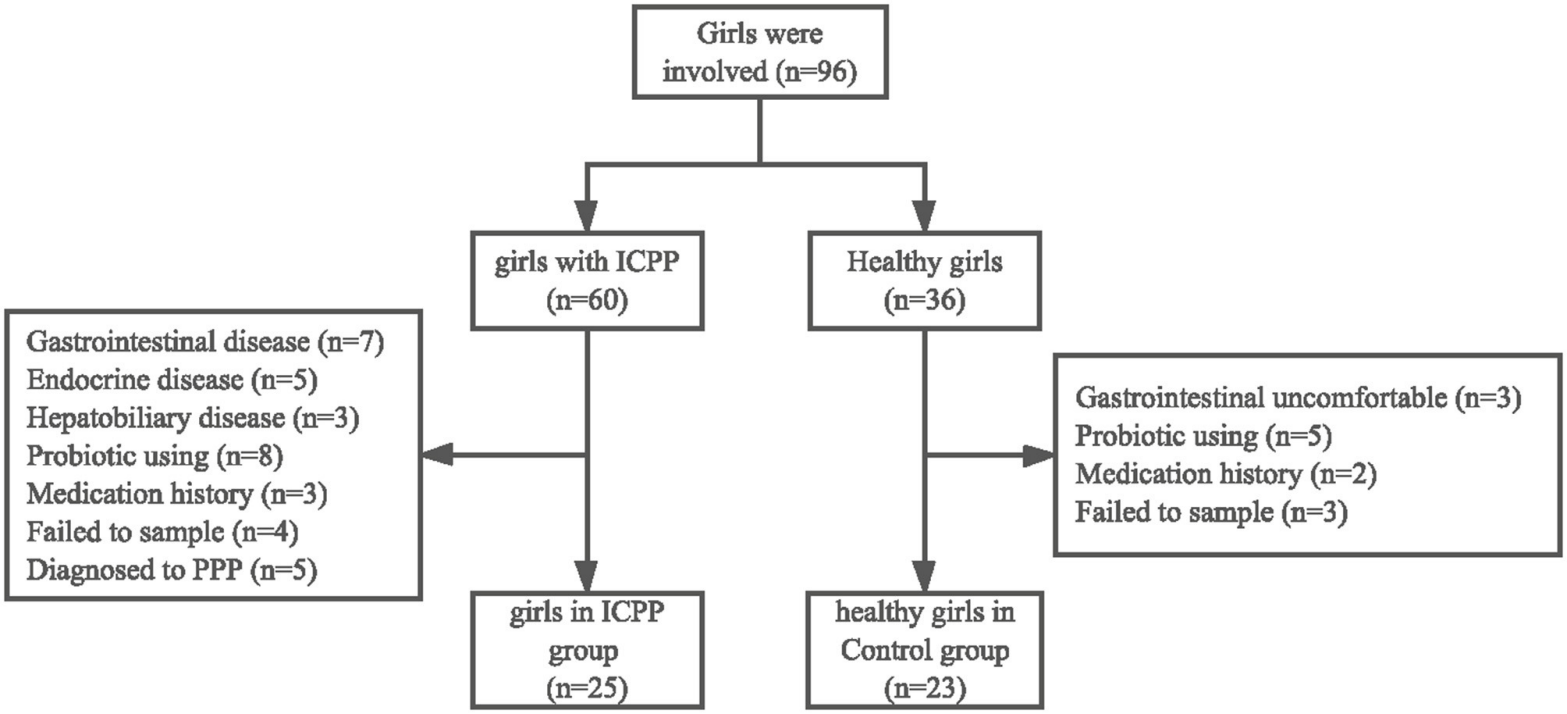
Flowchart illustrating the inclusion/exclusion of individuals in the study.

After applying the criteria described previously (Figure 1), a total of 25 girls with ICPP (ICPP group) and 23 healthy girls (Control group) were recruited for sample collection (Table 1, Supplementary Table 1). Power analysis showed that the power value was 0.524 when the effect size was 0.5 for a *t*-test with significance level of 0.05 (Supplementary Table 2). In the healthy group, 10 girls were pre-pubertal (age ≤ 8 years) and 13 girls were in puberty (age > 8 years). Four girls were diagnosed with obesity in the Control (*N* = 1) and ICPP (*N* = 3) groups (22). The high-quality paired-end reads from the 16S rDNA sequencing were connected, and each sample contained 42,127 ± 12,380 tags on average, which ranged from 11,137 to 57,400. After tag clustering, 590 OTUs were generated, and 173 ± 38.926 OTUs were yielded for each patients with ICPP, while 109 ± 33.131 OTUs were obtained for each healthy girl. After taxonomy annotation, a total of 145 genera and 204 species were identified for the study cohort.

**Table 1:** The clinical information of the ICPP and healthy girls.

**Characteristics**	**Control (*N* = 23)**	**ICPP (*N* = 25)**	***p*-value**
Age (mean ± SD, year)	8.462 ± 2.288	7.045 ± 1.08	0.024
BMI (mean ± SD, Kg/cm^2^)	16.733 ± 2.306	16.296 ± 1.865	0.574
FSH (mean ± SD, IU/L)	NA	18.085 ± 5.945	
LH (mean ± SD, IU/L)	NA	9.304 ± 4.588	
E2 (mean ± SD, pg/mL)	NA	26.28 ± 15.805	
TESTO (mean ± SD, ug/L)	NA	0.259 ± 0.236	
HCG (mean ± SD, IU/L)	NA	0.462 ± 0.856	
IR (mean ± SD)	NA	7.583 ± 2.761	
IGF-1 (mean ± SD, ng/mL)	NA	199 ± 88.795	
Bone Age (mean ± SD, year)	NA	8.442 ± 1.772	

*BMI, Body mass index; FSH, Follicle stimulating hormone; LH, Luteinizing hormone; E2, Estradiol; TESTO, Testosterone; HCG, Human Chorionic Gonadotropin; IR, Insulin Resistance; IGF-1, Insulin-like growth factor 1*.

### A Discrepancy in the GM Structure and Diversity Between the ICPP and Control Groups

The driving factors of microbiota composition were analyzed by PERMANOVA among the ICPP and Control groups. The results indicated that the disease status (FDR = 0.048) was the main driving factor compared to the age (FDR = 0.124) and BMI (FDR = 0.893). Therefore, the difference of microbiota in disease status (ICPP and Control groups) was analyzed in the next step.

At the genus-level, the top 10 genera in ICPP and Control groups were identified. Six genera were detected in the top 10 of both groups, namely, *Bacteroides, Faecalibacterium, Bifidobacterium, Blautia, Megamonas*, and *Parabacteroides*. Their relative abundances did not differ between the two groups (FDR > 0.05). In the top 10 genera of the ICPP group, the remaining four genera were *Ruminococcus* (FDR = 0.015)*, Prevotella* (FDR = 0.453)*, Gemmiger* (FDR = 0.013), and *Fusicatenibacter* (FDR = 0.123)*. Two* of these (*Ruminococcus* and *Gemmiger*) were significantly enriched in the ICPP group as compared with the Control group (FDR < 0.05). In the top 10 genera of the Control group, the remaining four genera were not significantly enriched in either group (FDR > 0.05), and these genera were *Lachnospiracea incertae sedis* (FDR = 0.078)*, Clostridium* XlVa (FDR = 0.280)*, Fusobacterium* (FDR = 0.555), and *Anaerostipes* (FDR = 0.546).

At the species-level, five species were observed in the top 10 of both groups, including *Faecalibacterium prausnitzii* (FDR = 0.834)*, Megamonas funiformis* (FDR = 0.218)*, Bacteroides ovatus* (FDR = 0.329)*, Bacteroides uniformis* (FDR = 0.770), and *Bacteroides plebeius* (FDR = 0.012). Of these, only *Bacteroides plebeius* was significantly enriched in the ICPP group as compared to the Control group (FDR < 0.05). In the top 10 species of the ICPP group, two of the remaining five species were significantly enriched in the ICPP group (FDR < 0.05), namely, *Bacteroides coprocola* (FDR = 0.047) and *Gemmiger formicilis* (FDR = 0.007). In the remaining five of the top 10 species of the Control group, only *Ruminococcus gnavus* was significantly enriched in the Control group (FDR = 0.019).

**Figure 2:**
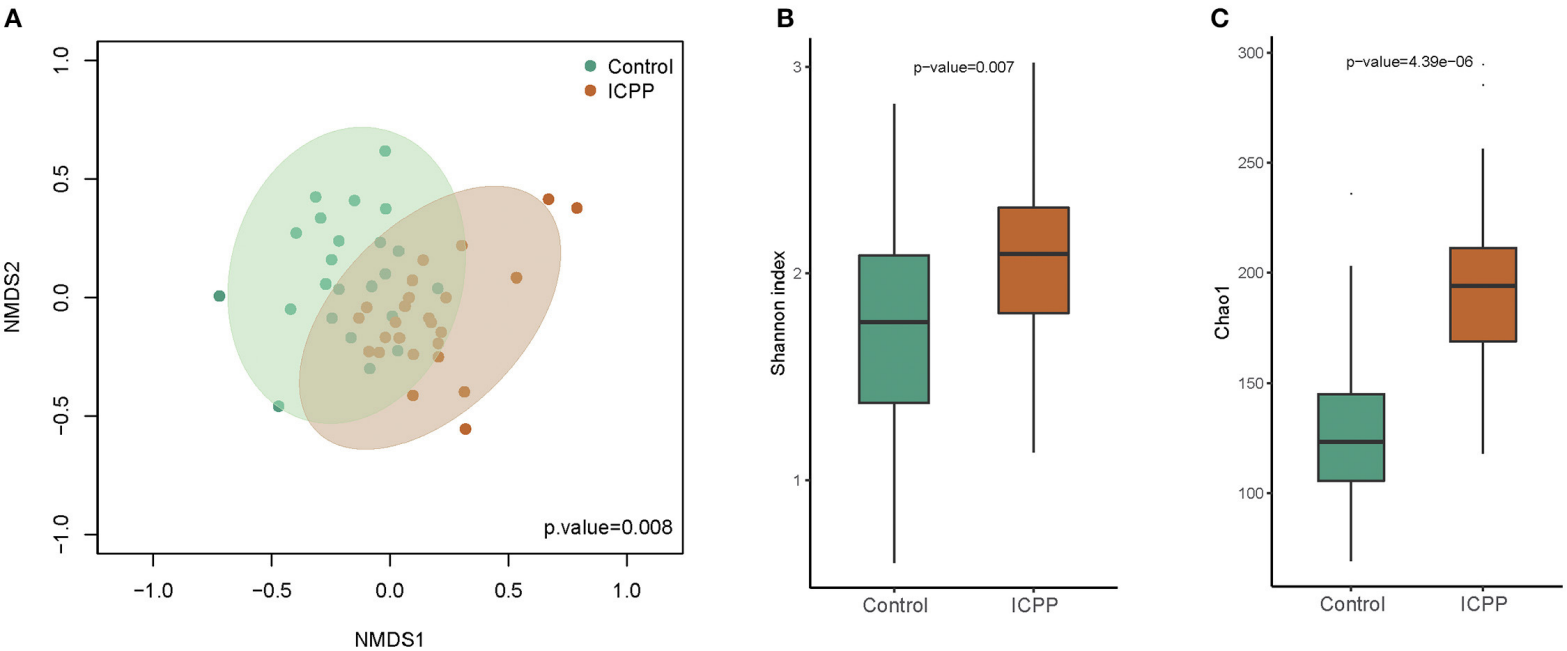
Non-metric multidimensional scaling (NMDS) distribution and microbiota diversity in ICPP and Control group. **(A)** NMDS analysis, the samples from ICPP group was clustered together, and they are separated from those of the Control group. **(B)** The diversity in the bacteria community was significant lower in the Control group than that in the Case group (*P* < 0.01). **(C)** The Chao indexed of gut microbiota was obviously lower in Control group than that in ICPP group (*P* < 0.01).

Power analysis of each genus illustrated that the power of the most different genera was more than 0.4 (Supplementary Figure 1). Furthermore, nMDS analysis illustrated that the GM samples from the ICPP group were clustered together and separated partly from the Control group (*P* = 0.008, Figure 2A). The clustered heatmap of the top OTUs and genera both illustrated the respective clustering tendency of the samples from the ICPP and healthy groups (Supplementary Figures 2, 3). Moreover, the ICPP group exhibited a significantly higher GM diversity than the Control group: the average value of the Shannon index was 2.079 ± 0.447 and 1.708 ± 0.512 for the ICPP and Control groups, respectively (*P* < 0.01, Figure 2B). The Chao1 index also showed higher diversity in ICPP group then that in Control group (*P* < 0.01, Figure 2C).

Collectively, the results documented that the main composition of GM in the ICPP group was similar to that in the Control group. Moreover, distinct microbes (genera or species) were identified between these two groups, and most were enriched in the girls with ICPP. These results suggested the potential relationship between ICPP and GM alteration.

### GM Biomarkers Containing Distinct GM Components Were Identified for ICPP Screening

**Figure 3:**
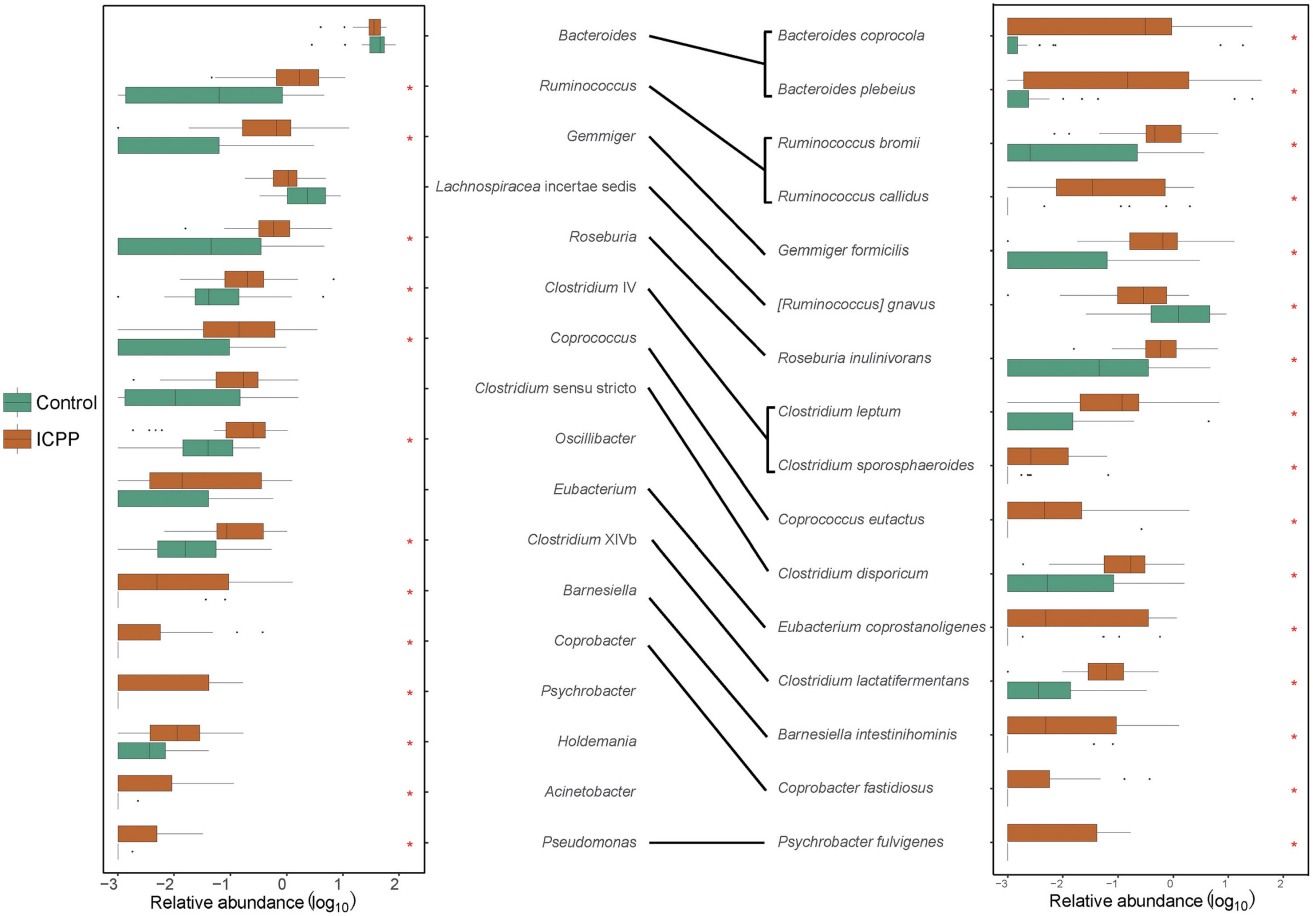
The discrepant genera and species between ICPP and Control group. Thirteen distinct genera and 16 discrepant bacterial species were found between ICPP and Control group. Most of them enriched in ICPP group. The asterisks stand for the *p* < 0.05.

To investigate the correlation between ICPP and the GM alteration, the significantly different microbes (genera or species) among the ICPP and Control groups were identified. At the genus-level, 13 genera were found to be visibly enriched in the ICPP group relative to the Control group (FDR < 0.05). These were *Ruminococcus, Gemmiger, Roseburia, Coprococcus, Clostridium sensu stricto, Oscillibacter, Clostridium XlVb, Barnesiella, Coprobacter, Psychrobacter, Holdemania, Acinetobacter*, and *Pseudomonas* (Figure 3). In the ICPP group, the relative abundances of the first three genera were more than 1% each time, and those of the remaining 10 genera were always <1%. By contrast, in the Control group, the relative abundance of all 13 genera enriched in the ICPP group was always <1% and two (*Coprobacter* and *Psychrobacter*) were not detected.

At the species-level, 16 different species were identified in the two groups (FDR < 0.05, Figure 3). Except for *Ruminococcus gnavus*, the remaining 15 species were enriched in the ICPP group. In the ICPP group, the relative abundances of five species (*Bacteroides plebeius, Bacteroides coprocola, Gemmiger formicilis, Ruminococcus bromii*, and *Roseburia inulinivorans*) were more than 1%. In the Control group, the relative abundances of the three discrepant species (*Bacteroides plebeius, Bacteroides coprocola*, and *Ruminococcus gnavus*) were more than 1%. In addition, two species (*Coprobacter fastidiosus* and *Psychrobacter fulvigenes*) were depleted in the Control group, which was consistent with the result from the genus-level.

The significantly different genera and species were highly consistent. However, some different results were also observed, such as the high relative abundance of *Ruminococcus gnavus* (2.477 ± 5.606%) in the Control group. We speculated that the result was caused by the low annotation rate of reads at the species-level (the reads used ratio was 59.38%).

**Figure 4:**
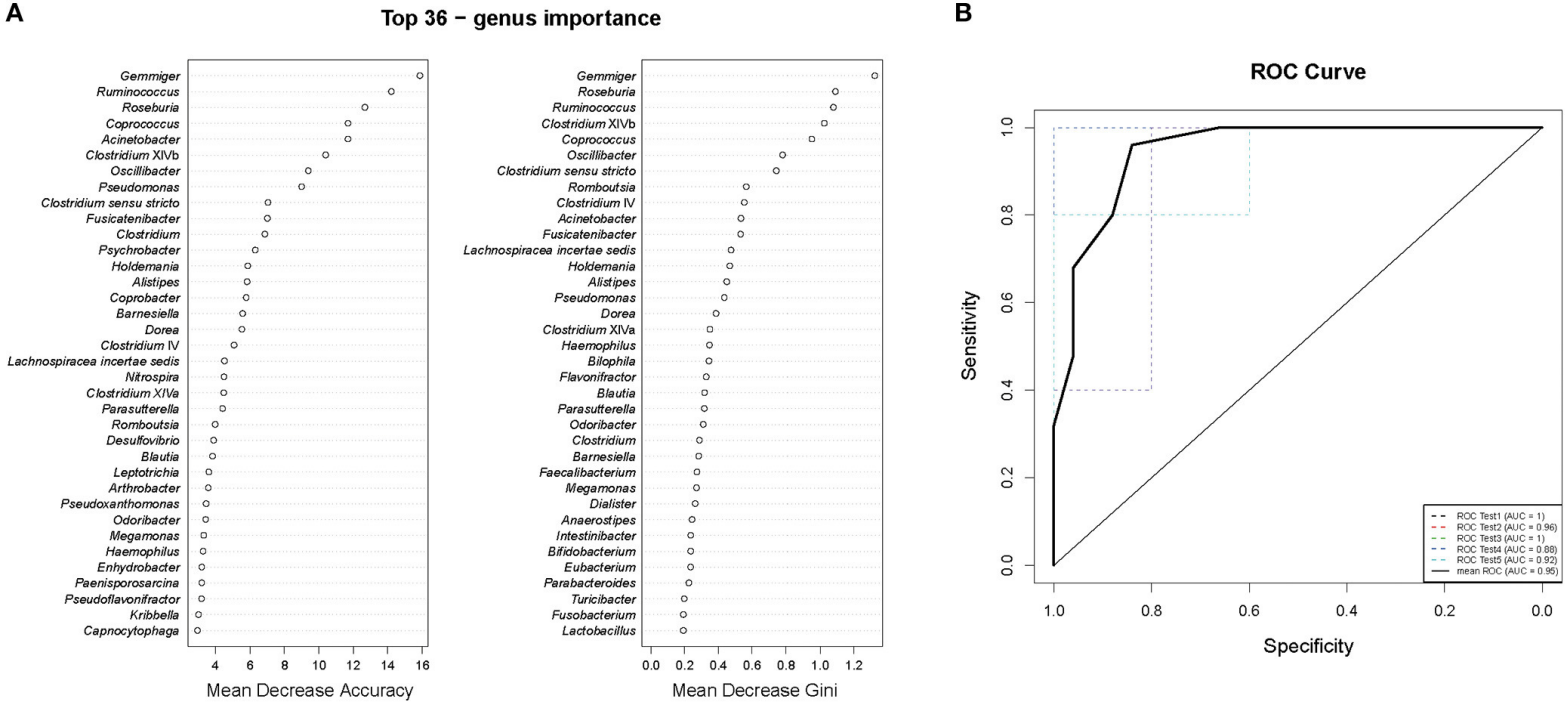
Gut microbiota (GM) biomarkers for classifying ICPP patients from healthy girls. **(A)** Mean Decrease Accuracy and Mean Decrease Gini of attributes as assigned by the random forest. **(B)** The accuracy of candidate GM biomarkers was verified with cross-validation. The AUC value was calculated and receiver operating characteristic curves (ROC) were drawn with five repeats.

To study the relationships between the ICPP and GM, we used genus information to construct a Random forest classifier for screening girls with ICPP. Figure 4A shows the first 36 features ordered by two specific importance measures: the Mean Decrease Accuracy and the Mean Decrease in Gini. We used these 36 features from the Mean Decrease in Gini for the training. The testing result showed that 36 features were able to differentiate patients with ICPP from healthy girls with high accuracy (AUC = 0.95, 95% CI 0.88 to 1, Figure 4B). These 36 candidate biomarkers included five genera with high Gini values: *Gemmiger* (Gini = 1.320), *Roseburia* (Gini = 1.086), *Ruminococcus* (Gini = 1.074), *Clostridium* XlVb (Gini = 1.020), and *Coprococcus* (Gini = 0.946). Furthermore, 11 of 13 significantly distinct genera between ICPP and the Control group were also identified as candidate biomarkers. The remaining two genera (*Coprobacter* and *Psychrobacter*) were excluded from the list of candidate biomarkers because of inferior Gini values.

### GM Functions Differ Between the ICPP and Control Groups

**Figure 5:**
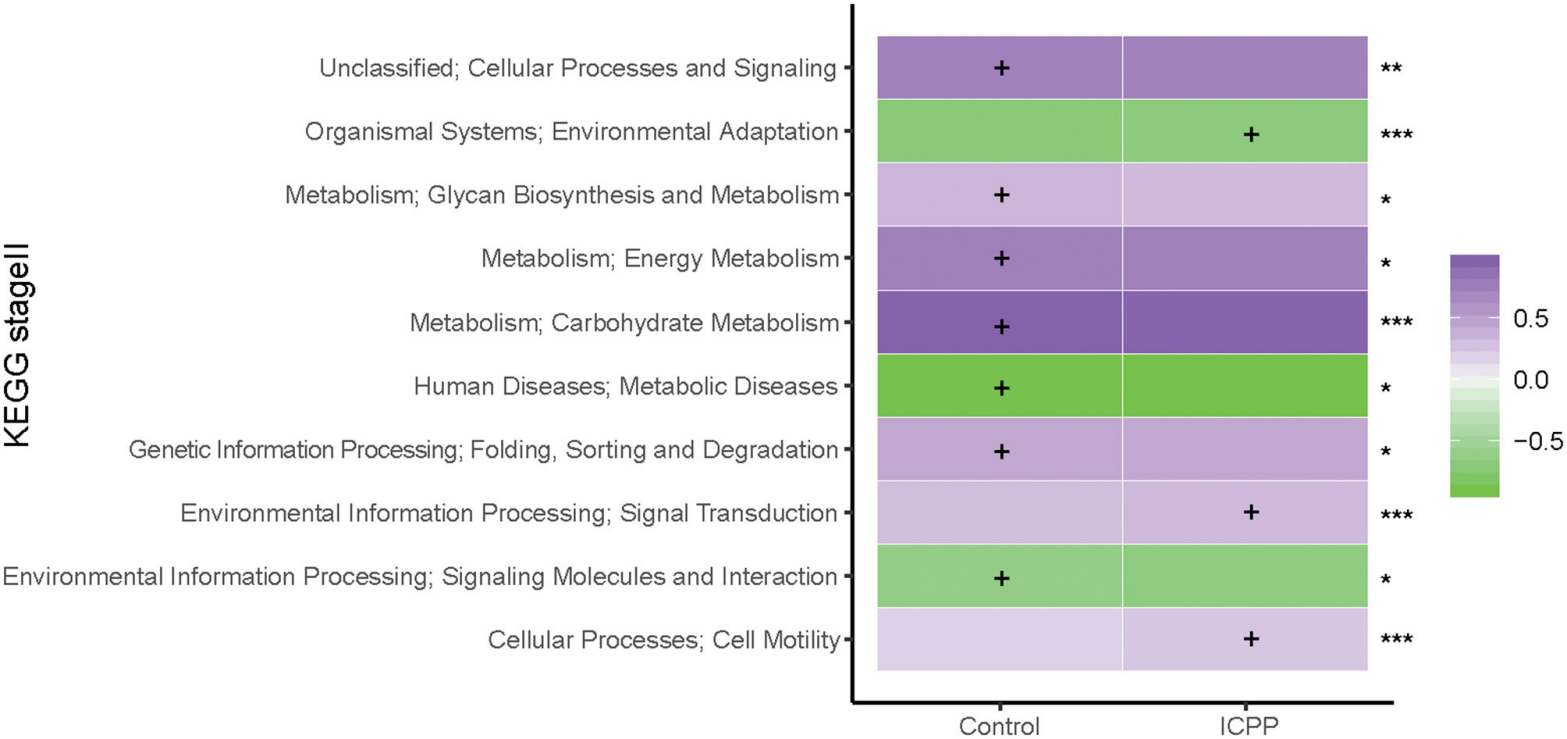
Relative abundance (log10 transformation) heat map of distinct KEGG categories in the ICPP and Control groups. Based on the functional classifications of the KEGG database, the functional categories between two groups were compared. FDR values indicated by asterisks on the right of Figure (***FDR < 0.001, **FDR < 0.01, *FDR < 0.05). The enrichment of functional category is indicated by cross.

To assess the influence of GM dysbiosis in ICPP, GM functions were explored. With PICRUSt prediction and KEGG annotation, a total of 37 functional categories were obtained. At the KEGG Orthology level II, we discovered 10 differentially enriched functional categories between two groups (FDR < 0.05, Figure 5). Three were enriched in the ICPP group, including Signal transduction (FDR = 0), Cell motility (FDR = 0), and Environmental adaptation (FDR = 0). In contrast, the remaining seven different functional categories were enriched in the Control group, including Carbohydrate Metabolism (FDR = 0), Energy Metabolism (FDR = 0.040), Cellular Processes and Signaling (FDR = 0.005), Folding, Sorting and Degradation (FDR = 0.037), Glycan Biosynthesis and Metabolism (FDR = 0.015), Signaling Molecules and Interaction (FDR = 0.037), and Metabolic Diseases (FDR = 0.037). Furthermore, the relative abundance of the functional categories in the Control group was higher than in the ICPP group. These results suggested that certain main GM functions changed in the ICPP girls along with the alteration of the GM.

### GM Taxonomies and Functions Were Associated With Several Clinical Indicators

To explore the relationships between GM and the occurrence of ICPP, pairwise correlations between the GM components and three crucial clinical indicators were estimated in the girls with ICPP. The clinical indicators included FSH, LH, and insulin resistance (IR). The results demonstrated that FSH was positively correlated with *Fusobacterium* (*r* = 0.633, *P* = 0.004), whereas LH was positively correlated with *Gemmiger* (*r* = 0.633, *P* = 0.004) and negatively correlated with *Romboutsia* (*r* = −0.465, *P* = 0.045) (Supplementary Table 3). Moreover, *Gemmiger, Ruminococcus, Megamonas*, and *Bifidobacterium* were positively correlated with IR (*r* > 0.400, *P* < 0.100, Supplementary Table 3). The associations between the GM functional categories and three clinical indicators were also investigated in the girls with ICPP. However, no reliable correlation was observed between the GM function and clinical indicators (*r* < 0.400 and *P* > 0.100, Supplementary Table 4). Furthermore, the correlation between genera and GM function analysis showed that LH-related *Gemmiger* was positively correlated with Environmental Adaptation and negatively correlated with Energy Metabolism and Signaling Molecules and Interaction. The IR-related *Ruminococcus* was positively correlated Environmental Adaptation and Cell Motility (Supplementary Table 5).

## Discussion

### GM Diybiosis in the ICPP Group Is Similar to That in the Obese Cohort

In this study, we mainly elucidated GM discrepancy between patients with ICPP and healthy girls without ICPP. The gut genera that were enriched in the ICPP group, such as *Ruminococcus Gemmiger, Oscillibacter*, and *Clostridium* XlVb, are similar to those that are associated with obesity. In a previous study, *Ruminococcus* had higher abundance in obese-prone rats or high-fat feeding mice compared with the control group (23). In clinical studies, *Ruminococcus* is enriched in individuals with obesity (24). Given that the presence of *Ruminococcus, Roseburia* and *Coprococcus* was positively correlated with methylamine (MA), which is a metabolite from choline, and these taxa might enhance the risk of obesity (25). Moreover, a previous study reported that *Gemmiger, Oscillibacter*, and *Clostridium* XlVb hold high relative abundances in obese-prone people as compared with normal-weight people (26). At the species level, we observed that *Ruminococcus bromii, Ruminococcus gnavus*, and *Clostridium leptum* were enriched in the girls with ICPP. *Ruminococcus bromii* has been found in obese populations, and could increase weight through the promotion of energy absorption and adipose tissue expansion (27). Furthermore, *Ruminococcus gnavus*, which was found to be enriched in obese rats, increased the occurrence of diverticulitis and is related to obesity (28, 29). Finally, *Clostridium leptum* was reported to be associated with human weight changes (30). These results documented that GM from patients with ICPP is similar to the GM in populations with obesity. The results may partially explain why girls who have precocious menarche are more likely to have obesity (31–33). We speculate that certain factors (such as diet, environment, and drug administration) would induce GM dysbiosis in these preadolescent girls, a process similar to that occurring in people with obesity. Eventually, these integrated factors (GM, metabolites, endocrine, and others) lead to the accumulation of fat in adipocytes and promote the onset of precocious puberty.

Additionally, we observed discrepant research results, namely, the finding that energy and carbohydrate metabolism categories of GM were enriched in the Control group in this study, functional categories also found to be enriched in the GM of people with obesity (11). We propose that the distinct enrichment of GM functional categories might be caused by diverse reasons: in girls with ICPP, GM dysbiosis would be induced by activation of the HPG axis; however, in people with obesity, the GM alteration would be triggered by accumulation of fat in the body.

### Enriched Bacteria in ICPP Group Might Induce Occurrence of ICPP Through Short Chain Fatty Acids Secretion

Leptin is an important metabolic peptide from adipocytes (34). Through the regulation of Kisspeptin (KISS1) neurons and the promotion of the pulsatile release of GnRH (35), leptin plays an important role in the onset of puberty in girls (36–39). Short-chain fatty acids (SCFAs, mainly butyrate and propionate) are correlated with leptin gene expression. SCFAs activates endogenous free fatty acid receptors (FFAR) such as FFAR2 and FFAR3 (40, 41), and these receptors are designated GPR43 and GPR41. Activated GPR41 may increase the expression of leptin and participate in the regulation of puberty (30). In our study, the girls with ICPP enriched microbes, including *Ruminococcus, Roseburia*, and *Ruminococcus bromii*, were associated with SCFA production. In the mouse model studies, *Ruminococcus, Roseburia*, and *Coprococcus* were negatively correlated with the monosaccharides derived from fecal metabolites, such as D-galactose, D-glucose, and D-xylose (25). Therefore, the three monosaccharide-consuming bacteria were potential promoters of SCFAs production (42, 43). On the species level, *Ruminococcus bromii* was positively correlated with the intake of resistant starch and generated SCFAs (44). Furthermore, *Ruminococcus callidus, Roseburia inulinivorans, Coprococcus eutactus, Clostridium sporosphaeroides*, and *Clostridium lactatifermentans* were related to SCFAs secretion (45–48), and they were also enriched in girls with ICPP. Therefore, we speculate that SCFAs-producing related bacteria are increased in the girls with ICPP to promote the expression of the leptin gene, activate the HPG axis through a high concentration of SCFAs, and lead to the onset of puberty.

In addition, we also identified 36 candidate microbial biomarkers that can be applied for the screening of patients with high accuracy. Interestingly, most of the microbial biomarkers with high Gini values were assigned to SCFAs-producing genera. These results suggest that there are potential associations between SCFAs-producing genera and the occurrence of ICPP.

Furthermore, estrogen-related bacteria reported in previous studies, such as *Clostridia* and *Ruminococcaceae*, were significantly different between ICPP and the Control groups (49). In addition, FSH- and LH-associated genera were also identified in our study. However, the mechanism of involvement of these genera in the secretion of estrogen, FSH, or LH, and in the ICPP progress, is still unclear and needs further study.

Generally, the characteristics of GM dysbiosis were detected in girls with ICPP in our study. We speculate that a GM alteration could induce an increase of SCFAs-producing bacteria, which might up-regulate the expression of leptin and increase the secretion of GnRH. Ultimately, the HPG axis is activated, and ICPP occurs. We also identified GM biomarkers for clinical prediction of ICPP, and it should be possible to detect more accurate GM biomarkers than these with larger population studies.

A number of limitations should be noted in our study: (1) we did not administer and analyze dietary questionnaires; (2) serum leptin levels were not tested; and (3) fecal metabolomics were not investigated.

In summary, this research discovered GM alterations in girls with ICPP, and it provided foundations for the prediction and prevention of ICPP through the analysis of the GM.

## Data Availability Statement

The datasets generated for this study are included in the article/Supplementary Files. The row sequencing reads have been deposited in the GenBank repository under the accession number: PRJNA511633.

## Ethics Statement

The studies involving human participants were reviewed and approved by Shenzhen Maternity and Child Health Hospital Ethics Committee number: (2018)216. Shenzhen Maternity and Child Health Hospital. Written informed consent to participate in this study was provided by the participants' legal guardian/next of kin. Written informed consent was obtained from the individual(s), and minor(s)' legal guardian/next of kin, for the publication of any potentially identifiable images or data included in this article.

## Author Contributions

JZ, MQ, XL, and PL performed the sampling and information collection. JL and MH prepared the DNA. DL, XF, YL, and ZY performed the bioinformatics analysis in this work. ZY and WD interpreted the analysis results and wrote the paper. XX, XL, YZ, YL, and ZY guided statistics analysis and polished the article. GD, WD, and SG managed the project.

### Conflict of Interest

XF, DL, YL, and WD were employed by the company Wehealthgene Co. Ltd. The remaining authors declare that the research was conducted in the absence of any commercial or financial relationships that could be construed as a potential conflict of interest.

## Supplementary Material

Supplementary Figure 1 Power analysis for all genera.

Supplementary Figure 2 Clustered heatmap of top 20 OTUs.

Supplementary Figure 3 Clustered heatmap of top 21 genera (relative abundance > 0.5%).

Supplementary Table 1 Sample information.

Supplementary Table 2 Power effect analysis.

Supplementary Table 3 The correlation between genera and three clinical indicators.

Supplementary Table 4 The correlation between KEGG functional categories and three clinical indicators.

Supplementary Table 5 The correlation between genera and KEGG functional categories.
