# Supplementary figures and images for: The Association of Gut Microbiota With Idiopathic Central Precocious Puberty in Girls
Source: Front Endocrinol (Lausanne). 2020 Jan 22;10:941. doi: 10.3389/fendo.2019.00941 (PMC6987398; doi:10.3389/fendo.2019.00941)

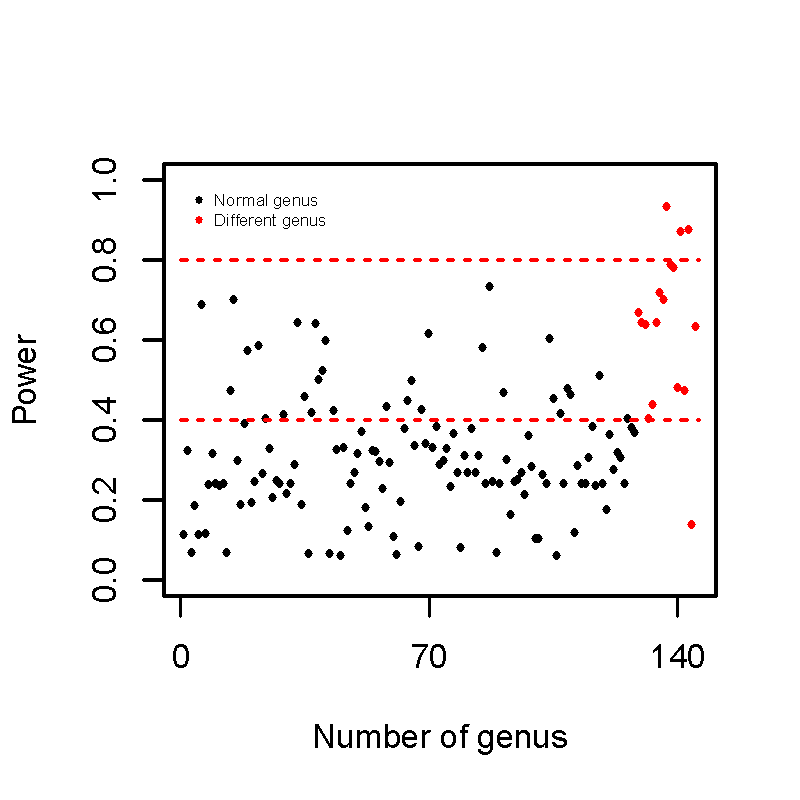

Supplement: Supplementary Figure 1 — Power analysis for all genera. [file Image_1.TIFF]

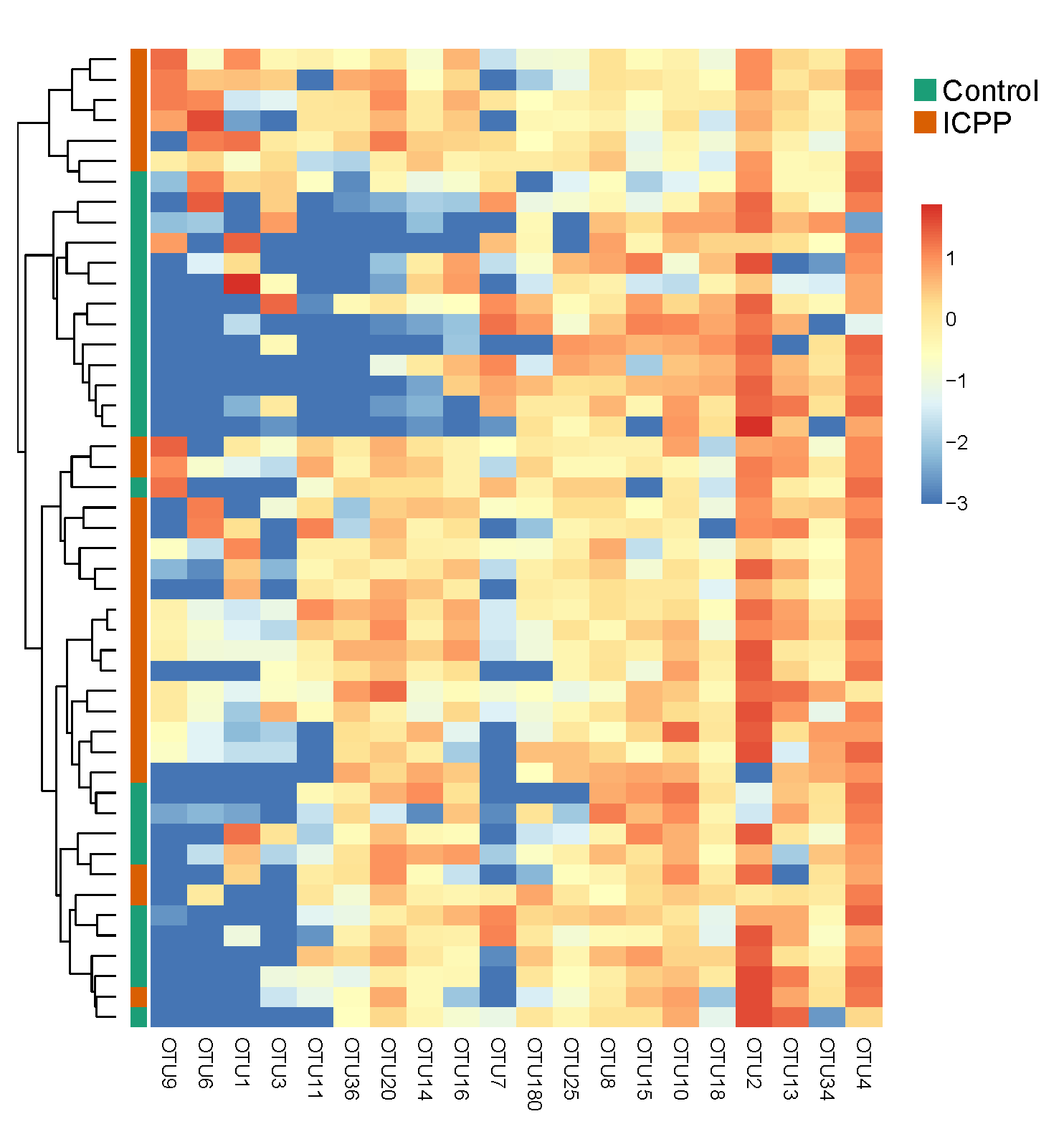

Supplement: Supplementary Figure 2 — Clustered heatmap of top 20 OTUs. [file Image_2.TIFF]

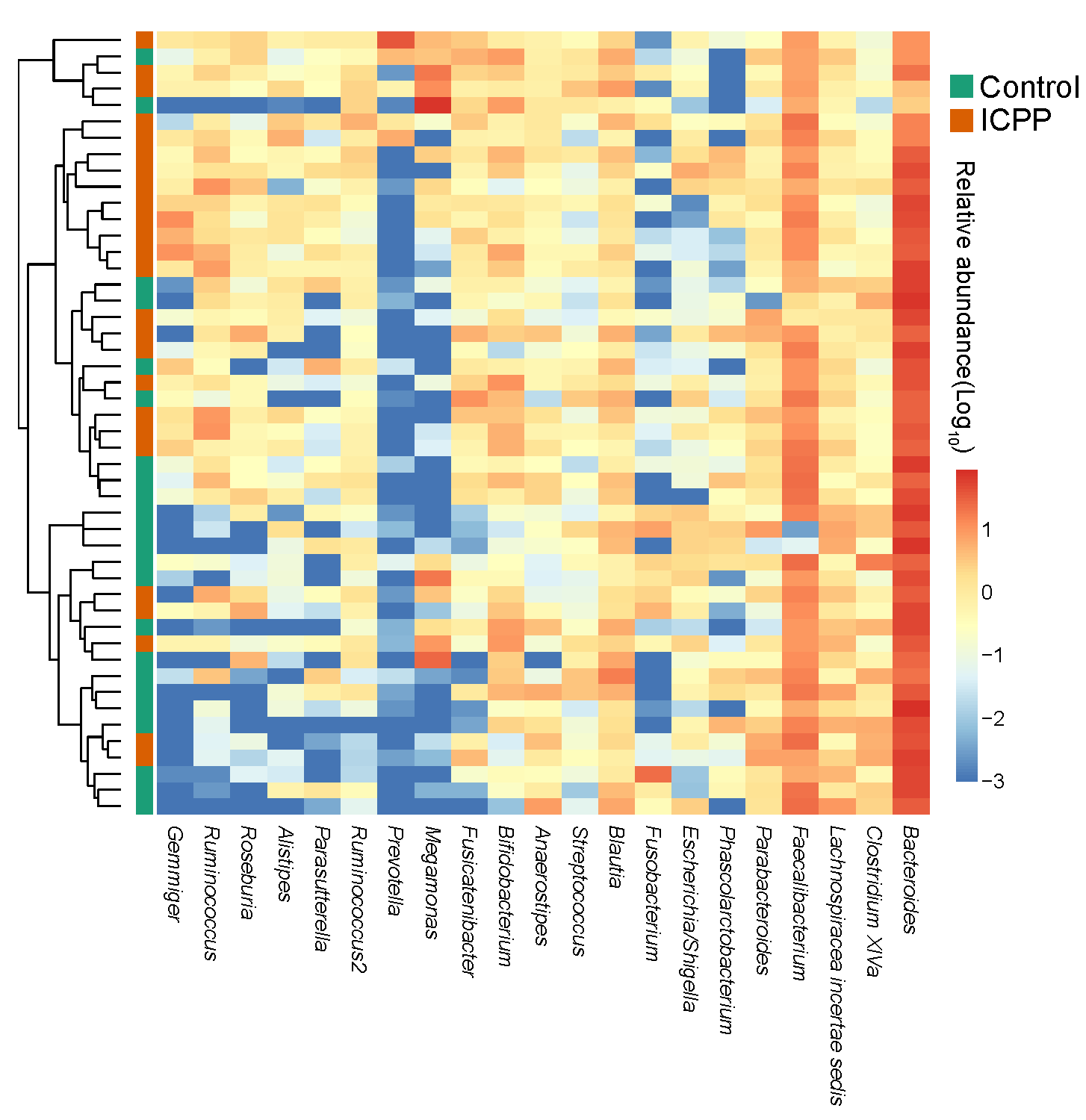

Supplement: Supplementary Figure 3 — Clustered heatmap of top 21 genera (relative abundance > 0.5%). [file Image_3.TIFF]
